# IMP^2^ART: development of a multi-level programme theory integrating the COM-B model and the iPARIHS framework, to enhance implementation of supported self-management of asthma in primary care

**DOI:** 10.1186/s43058-023-00515-2

**Published:** 2023-11-13

**Authors:** Liz Steed, Jessica Sheringham, Kirstie McClatchey, Vicky Hammersley, Viv Marsh, Noelle Morgan, Tracy Jackson, Steve Holmes, Stephanie Taylor, Hilary Pinnock

**Affiliations:** 1 Wolfson Institute of Population Health, Barts and the London School of Medicine and Dentistry, Queen Mary University of London, London, UK; 2 Department of Applied Health Research, University College London, London, UK; 3 Asthma UK Centre for Applied Research, Usher Institute, The University of Edinburgh, Edinburgh, UK; 4 The Park Medical Practice, Shepton Mallet, UK; 5 Severn School of Primary Care, Health Education England (South West), Bristol, UK

**Keywords:** Asthma, Supported self-management, IMP^2^ART, Implementation, Theory

## Abstract

**Background:**

Supported asthma self-management, incorporating an asthma action plan and annual clinical review, has been recommended by UK/global guidelines for over three decades. However, implementation remains poor, as only around a third of individuals receive basic asthma care, according to the UKs leading respiratory charity Asthma and Lung UK. A systematic review of implementation studies recommended that a whole systems approach targeting patients, healthcare professional education, and organisations is needed to improve implementation of supported asthma self-management in primary care. The IMPlementing IMProved Asthma self-management as RouTine (IMP^2^ART) is a national Hybrid-II implementation cluster randomised controlled trial that aims to evaluate such an approach. This paper describes the development of the implementation strategy for IMP^2^ART with particular focus on the integration of multiple level theories.

**Methods:**

The Medical Research Council design and evaluation of complex interventions framework and the Person-Based Approach to intervention development were used as guidance for stages of strategy development. Specifically, we (i) set up a multidisciplinary team (including practicing and academic clinicians, health psychologists, public health and patient colleagues), (ii) reviewed and integrated evidence and theory, (iii) developed guiding principles, (iv) developed prototype materials, and (v) conducted a pre-pilot study before final refinement.

**Results:**

The implementation strategy included resources for patients, team-based and individual healthcare professional education, practice audit and feedback, and an asthma review template, as well as a facilitator role accessible to primary care practices for 12 months. The synthesis of the integrated Promoting Action on Research Implementation in Health Services (iPARIHS) and Capability, Opportunity, Motivation and Behaviour (COM-B) frameworks led to an evolved framework bringing together important implementation and behaviour change elements which will be used as a basis for the study process evaluation.

**Conclusions:**

A description of rigorous implementation strategy development for the IMP^2^ART study is provided along with newly theorised integration of implementation and behaviour change science which may be of benefit to others targeting implementation in primary care.

**Trial registration:**

ISRCTN15448074. Registered on 2nd December 2019.

**Supplementary Information:**

The online version contains supplementary material available at 10.1186/s43058-023-00515-2.

Contributions to the literature

- This article illustrates integration of multiple theories to inform a comprehensive multi-level programme theory for implementing supported self-management for asthma in primary care
- The article responds to recent calls to build on the iPARIHS implementation framework, by embedding COM-B within the recipient element
- The article proposes ways in which a process evaluation can iteratively inform and advance development of theory within the field.

## Background

The challenge of interventions of proven effectiveness in randomised trials not translating into clinical practice is at the crux of implementation science. Asthma supported self-management, which incorporates an asthma action plan and regular annual review by a healthcare professional [1], is a case in point. Supported self-management has been shown to be cost-effective, reduce hospitalisations, emergency department attendances, and unscheduled healthcare; improve markers of asthma control and patient quality of life in numerous trials and systematic reviews [2–4]. Consequently, for the past three decades, both national and international guidelines have recommended such care for individuals with asthma [5, 6] yet implementation remains poor. International surveys typically report only between one third and one half of people with asthma own an action plan and less than 40% receive the three key aspects of basic asthma care when defined as including an annual review, owning an action plan, and having an inhaler check [7–11]. Moreover, routine primary care data from our developmental work revealed that only 6% had a coded record of being given an action plan [12]. Tackling barriers to implementation of asthma supported self-management is therefore a priority within asthma care [13]. Further, given asthma is a common, non-communicable illness affecting approximately 262 million people internationally [14] and causing 455,000 deaths annually [15] (many of which could be avoided if symptoms were controlled with proper management) [13, 16, 17], the problem is a challenge for healthcare worldwide.

Meta-reviews exploring implementation of supported self-management in chronic illness have previously indicated that whilst patient education, professional education, and organisation strategies are all essential for successful implementation of supported self-management, rarely are they integrated and evaluated together [18, 19]. A multifaceted and multidisciplinary approach within asthma which provides resources for patients, educates, and motivates healthcare professionals, and does so within the context of an organisation which actively endorses supported self-management, has therefore been called for [19]. As such, a whole systems approach is needed and is currently at the heart of asthma health service recommendations within the UK [20].

A challenge when conducting such work however becomes how to incorporate and integrate sound theoretical knowledge at multiple levels, with the individual, team, and organisational implementation strategies being informed by often complementary but potentially different theories. Birken et al. [21] suggest that use of multiple theories can help researchers address multiple study purposes, but also caution that in some cases multiple frameworks may add unnecessary complexity and redundancy. A key consideration when selecting implementation theories is whether they also work in harmony with the original intervention theory to produce a cohesive whole. A potential benefit of theory integration may be enabling design of process evaluations to fully explore the mechanisms by which both the implementation and intervention are posited to work, and thus contribute to progress in the field. This responds to calls for the need to use empirical data from process evaluations to refine implementation theory [22].

The aim of this paper is to show how we developed an integrated programme theory, and how this informed both the implementation strategy and the implications for the process evaluation of a large, UK wide Hybrid type II cluster randomised trial IMP^2^ART (IMPlementing IMProved Asthma self-management as RouTine) [23]. Here we describe the methods and results of our theoretical integration, how this informed the evolving implementation strategy, and the final strategy following a feasibility pre-pilot trial of the strategy. We do not aim to report results of the main trial which tests the impact of a whole systems implementation strategy that embeds supported asthma self-management in primary care, compared with usual care on: unscheduled care (clinical outcome) and ownership of an action plan (implementation outcome).

Similarly, findings from a process evaluation which will explore how supported self-management was implemented (or not) to aid interpretation, inform scaling up and sustainability will be published separately.

This article aims to generate learning around the implementation of supported self-management in primary care more generally, as this is a critical element in the management of most chronic illnesses.

## Method

**Fig. 1:**
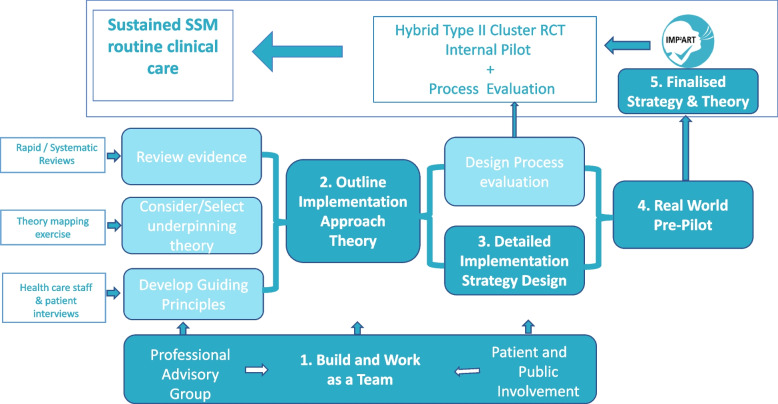
The development and evaluation process of the IMP^2^ART Implementation Strategy. Evidence review, theory mapping and guiding principles informed specification of outline of the implementation approach which was then translated into detailed design of the implementation strategy (and concurrent process evaluation design) followed by testing in a pre-pilot, refinement and evaluation in a cluster randomised controlled trial with internal pilot

Design of the implementation strategy was informed by MRC guidance [24] and the person-based approach to intervention development [25] and is outlined in Fig. 1. Importantly, each step was used iteratively to inform the next step with repeated cycling between steps where needed.

The study was supported by grants from the National Institute of Health and Care Research (NIHR) in the UK, one understanding the context and challenges of implementing asthma self-management (NIHR RP-DG-1213–10,008) and one for development and evaluation of the strategy (NIHR RP-PG-1016–20008). The Asthma UK Centre for Applied Research supported some additional theoretical work (AC-2012–01).

### Building a team

At the outset of the programme of work, a stakeholder map was created of individuals involved in asthma self-management in primary care, and representatives from each of these disciplines were invited to form the initial study team. At key points (i.e. when initial theory and guiding principles were identified), the map was reviewed, and further members were invited to join the team as needed. An implementation strategy development working group was set up to ensure multidisciplinary input into IMP^2^ART. In addition, working groups with expertise around theory and process evaluation (led by LS and JS), a patient and public involvement group (led by TJ and NM) and a professional advisory group (led by SH) were set up.

The function of the team was to bring tacit knowledge of the issues that may need to be addressed and serve as a starting point for directions of travel, some of this occurring at the stage of funding applications and developed iteratively as the development progressed.

### Outline implementation approach and theory

#### Review of evidence

In line with the whole systems approach, implementation components were planned to target patients, healthcare professionals and organisational structures. Preliminary work searched for literature reviews in these defined areas to ensure research efficiency and build upon the existing evidence base [24]. A taxonomy of important components of support for asthma self-management derived from the PRISMS study [26] and considered in the asthma context [19] was available; however, a gap in the content and delivery of healthcare professional education was identified. A systematic review of professional education interventions in asthma or other long-term conditions [27] was therefore conducted in accordance with Cochrane methodology. This scoped the availability of education packages and coded interventions using the Theoretical Domains Framework [28], to understand whether certain theoretical constructs were important to include.

Literature suggested audit and feedback led to a “potentially important” improvement in practice [29], and our initial qualitative work suggested a pivotal role for templates in determining the content of asthma reviews [30]. We undertook a systematic review to inform the optimal design for a template to promote patient-centred care [31] and scoped the context related to supported self-management in national and international guidelines [32].

#### Use of theory

In addition to building on previous evidence, the MRC complex intervention framework [24] and related guidance on intervention development [33] strongly recommend underpinning intervention development with theory. However, the use and application of theory has become increasingly complex with Nilsen [34] and Kislov et al. [22] both reporting theory descriptors at multiple levels. At the outset, we conceived there would need to be theory informing the content of strategies for patients, healthcare professionals (individuals and teams) and the organisation, as well as an overarching implementation theory. We expected that patient and healthcare professional strategies would be influenced by the COM-B model [35] which specifies that Behaviour at both individual and group levels is influenced by Capability (physical, e.g. dexterity and psychological, e.g. memory), Opportunity (physical, e.g. resources and social, e.g. whether important others support the behaviour) and Motivation (both automatic, e.g. habits and reflective, e.g. beliefs). This model is recommended for interventions targeted at behaviour change in national guidance [36]. From our initial exploratory work in primary care practices [30], we also anticipated that organisational strategies would be informed by routinisation theory [37], whereby a behaviour is repeated frequently in the same context until it becomes a routine (or habit), requiring less cognitive power. We envisaged that by understanding where there are weaknesses in either a patients’ or healthcare professionals’ capability, opportunity or motivation to self-manage or support self-management, we would be able to target this with a specific intervention, e.g. education, provision of resources, or motivational discussion. By working with teams over a year to provide behaviours supporting self-management, e.g. discussing asthma action plans at every asthma consultation, this would then become routine practice.

In order to check that these were the most appropriate theories to integrate into an overarching programme theory, and to ensure that evolving implementation strategies were fully theoretically informed, three team members with extensive theoretical expertise in behaviour change, implementation science and organisational change (LS, JS and KM) undertook a mapping exercise (see supplementary file 1). Theories were reviewed and both theories and strategies were mapped on to each other.

The mapping was subsequently presented to the process and intervention development teams with feedback integrated into the developing programme theory. In addition, an implementation logic model was developed in collaboration with the team.

#### Develop guiding principles

To elicit understanding of what patients and healthcare professionals saw as their most important needs for supporting self-management, and the key principles of the implementation strategy, qualitative work had been conducted with both professional and patient groups. For the professional group, 23 general practitioners, seven nurses and three administrative staff, across 14 different primary care settings, agreed to participate in either a single focus group or interview. The topic guide explored views on existing routines for supported self-management, barriers to its provision and possible solutions. Thematic analysis was conducted on anonymised transcripts. Full methods have been described previously [30].

For the patient interviews, 49 participants (including 45 patient and four parent/carers) from ten general practices participated and expressed views on understanding and conduct of self-management, how behaviours had developed, barriers to self-management and ways to overcome this. Analysis used an interpretative phenomenological approach and was conducted with consultation of a multidisciplinary steering group. This has been described in full [38].

Key findings from both sets of qualitative work were presented to the intervention development team who then reached consensus on the guiding principles.

### Develop detailed implementation design

Having completed the above steps preliminary patient, professional and organisational components were identified that were in line with the evidence, theory and guiding principles. For each component, a range of prototypes was developed bearing in mind the theoretical underpinnings and other relevant considerations. For example, the educational modules were informed by andragogy (adult learning) and e-learning methodologies (see [39] for further details).

Prototypes were reviewed by the implementation strategy development group as well as the patient advisory and professional advisory group with iterative feedback and re-design (see supplementary file 2 for greater detail on strategy).

### Pre-pilot

A real-world pre-pilot was conducted to evaluate how the implementation strategies ‘hung’ together as a whole. Four general practices were identified and provided with the prototype implementation strategy.

Debriefing was used to gain initial reflections from the facilitator on acceptability. Interviews were conducted for individuals completing education modules and reviewing other resources.

### Finalising the implementation strategy and programme theory

Following the pre-pilot, a workshop (August, 2019) was held with the implementation strategy development group to consider any changes that needed to be made. Potential changes were presented to the team and the MoSCow approach [40] was used to agree whether changes were needed.

In March 2020, just prior to commencement of the planned internal pilot (April 2020), the global COVID-19 pandemic hit. This required the majority of non-COVID research to pause and created a rapidly changing climate for future implementation of healthcare. It quickly became apparent that healthcare provision would make increasing use of technological and remote approaches [41, 42]. The IMP^2^ART project therefore used the enforced pause in recruitment as an opportunity to review the implementation strategy, for the changing healthcare context. All strategies that had been agreed were reviewed for potential optimisation for a future where remote and digital delivery of healthcare is more prevalent. Where optimisation was possible, steps were reinitiated and the whole implementation strategy was updated (supplementary file 2 indicates changes made to the implementation strategy as well as full detail in the trial protocol [23]). One final opportunity to review the implementation strategy was the internal pilot although this was designed primarily to test trial processes.

The final programme theory was outlined and presented to the working group. This was used as an important element for further designing the process evaluation.

## Results

### Building a team

From the outset, a multidisciplinary team of collaborators including patient colleagues, primary care clinicians and academics, implementation researchers, educationalists (Education for Health), health psychologists, trial methodologists, health economists, and a ‘not-for-profit, social enterprise’ (Optimum Patient Care) was formed. Early work further highlighted the importance of organisational change and facilitators as enablers of change and hence additional team members representing these areas were included (VM). In addition to individual team members, the project was embedded within the Asthma UK Centre for Applied Research, (https://www.ed.ac.uk/usher/aukcar) an initiative bringing together expertise from across the UK designed to increase collaboration and optimise delivery and impact of asthma research.

### Outline implementation approach and theory

#### Tacit knowledge

Aligned with the Quality and Outcome annual targets [43], it was deemed that the implementation strategy should include 12 months of active facilitation followed by a 12-month phase for embedding and adapting to fully incorporate audit and feedback, templates, healthcare professional education and patient resources to individual practices.

#### Review of evidence

The literature review of professional education interventions concluded that inclusion of strategies such as endorsement by local opinion leaders, making education inter-professional and having clear reference to guideline recommendations would be useful. Theoretical domains shown to be potentially useful to address included *‘*social influences, e.g. social support’; ‘environmental context and resources’ e.g. provision of an action plan’; ‘behavioural regulation’, e.g. self-monitoring of peak flow; ‘beliefs about consequences’, e.g. that adherence to medication will improve asthma control; and ‘social/professional role and identity’ such that several members of the team see they have a role in self-management not simply the ‘asthma nurse’ [27].

The systematic review of templates in long-term conditions [31] concluded that templates have both advantages and disadvantages. Whilst they can be a helpful prompt in consultations and guide priorities, ensuring templates facilitate patient-centred care, for example by explicitly asking about the patient’s agenda, is important.

The review of audit and feedback suggested that optimal feedback would be received regularly (monthly), brief, use other general practices as comparators and include different modes of data presentation.

#### Theory

##### Identified theory

As the preliminary work had already made use of approaches from the Behaviour change wheel (i.e. the Theoretical Domains Framework), LS and KM recommended that the Capability, Opportunity, Motivation Framework [35] would be a useful model from behaviour change science to guide further development of the implementation strategy. It has the advantage that it was designed to be applied for interventions at multiple levels of the healthcare system from policy change to individual-level behaviour change and therefore resonated well with the objective of IMP^2^ART to facilitate change at individual, team and organisational levels.

In reviewing the organisational change literature (e.g. [37, 44]), the team felt it would be important to tailor implementation strategies to fit in with the routines of an individual practice. However, it became apparent that routinisation theory may be too narrow and that other related organisational constructs such as culture (for example how hierarchical a practice is, or open to new initiatives) and resources should also be considered in IMP^2^ART. It was agreed that whilst it may not be feasible for IMP^2^ART to substantially alter culture or resources [45], understanding how such constructs could impact on implementation success would be important.

As discussions around the implementation strategy evolved, it became apparent that the role of a facilitator (i.e. an individual linked to a practice to support and tailor IMP^2^ART resources) to support enactment of change was pivotal. This led one of the theory stakeholders (JS) to present the team with another implementation theory—the integrated Promoting Action on Research Implementation in Health Services (iPARIHS) framework [46]. iPARIHS holds that for successful implementation there are four key constructs (i) Innovation—which revolves around the evidence and knowledge sources to be applied, but with adaptation where appropriate dependent on the local setting, i.e. supported self-management with use of IMP^2^ART resources, (ii) Recipients—which speaks to the stakeholders (both individuals and teams, e.g. the patient or healthcare professional and the primary care team) involved in the implementation and their attitudes, beliefs and behaviours that impact the implementation, (iii) Context—which reflects the micro, meso and macro levels by which context influences change. It is defined in iPARIHS in terms of resources, culture, leadership and orientation to evaluation and learning. The final and key construct is ‘facilitation’ which is seen as the active ingredient that works with the recipients within their context to implement the innovation. The role of the facilitator is to understand the interacting factors and tailor input accordingly. Given the importance of tailoring implementation strategies according to the complexity that is primary care practice, this resonated strongly with the study aims. In addition, the broader understanding of context within iPARIHS was felt to be helpful and this reinforced the decision to underpin implementation with iPARIHS.

##### Theoretical integration

**Fig. 2:**
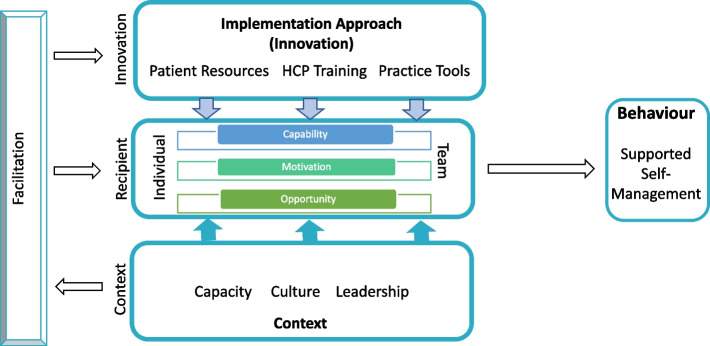
Integration of the iPARIHS and COM-B in optimising supported self-management in primary care. The IMP^2^ART implementation strategy, combining knowledge around the benefits of supported self-management and tools to embed this (Innovation), is hypothesised to impact supported self-management (Behaviour) via primary care health care professionals and their patients (Recipients) targeted according to their capability, motivation and opportunity, with Facilitation acting as a catalyst to change and a tailoring source to support adaptations according to context, recipient needs and prior use of innovation

Having identified the primary theories to guide the implementation process, a key aim was to advance the field of implementation science by proposing an integrated model representing the coming together of behaviour change, implementation and organisational constructs and theory, which we could allow them to be interrogated, revised and developed in the light of our empirical process evaluation findings as recommended [22]. Figure 2 is our proposed integrated model.

In this model, behaviour (i.e. implementation of supported self-management) is enacted by recipients (individual or team, healthcare professional or patient), with the support of the tailored implementation strategy (the innovation) that maximises both individuals and team motivation (willingness to perform a behaviour), capability (whether someone has the physical, e.g. dexterity to use an inhaler or psychological ability, e.g. recall) and opportunity (either social such as feeling their healthcare professional endorses the behaviour or practical such as having enough appointments to see patients) through practice-specific assessment and support from the facilitator who accounts for and overcomes challenges for individual practices within their given context.

#### Guiding principles

Table 1 shows the key findings from each of the preliminary studies to understand the needs of key stakeholders and the current evidence base. From these, and in conjunction with the outlined theory, three main guiding principles were articulated and thus were used as core components of the implementation strategy. A fourth was added later due to COVID-19.

1. Emphasising a team approach
2. Incorporating strategies within individual practice routines, encouraging adaption to promote a tailored approach
3. Ensuring a patient-centred approach to care throughout
4. Supporting practices in adapting their care to either partial or entire remote delivery

**Table 1:** An overview of preliminary studies with key findings for the implementation strategy

Publication	Methodology	Key findings for implementation strategy
**Patient**		
Daines et al. 2020 [38]	Interviews and focus groups with 45 patients and 4 carers. Interpretative phenomenological analysis	Self-management behaviours were both automatic (habitual) and reflexive (cognitively driven) when symptoms changed. Support was welcomed, particularly when diagnosed or when symptoms were unstable
McClatchey et al. 2021 [47]	Survey of 95 individuals with asthma. Descriptive analysis	Patients received most of their information from their General Practitioner/Practice Nurse or websites but there was a desire for personalised information and further direction from healthcare professionals
**Professional**		
Morrow et al. 2017 [30]	Interview and focus groups with 33 health care professionals Thematic analysis	Need to emphasise evidence for benefit of supported self-management, improve team work through team based education, include remote working and fit within current routines, including improved templates
McCleary et al. 2018 [27]	Systematic review with 18 included studies. Narrative synthesis	Effective initiatives more often included guideline based, involved, local opinion leaders and included inter-professional learning
**Organisational**		
Morrissey et al. 2021 [31]	Systematic review with 12 qualitative and 14 quantitative studies. Narrative synthesis	Templates are well used and act as useful reminders but risk the professional agenda taking precedence over that of the patient. Future templates should be designed to promote patient-centred care
McClatchey (Personal communication)	Rapid review of audit and feedback characteristics	Regular feedback with individually meaningful comparators which include varied visual presentations are likely to be more effective
Kinley et al. 2021 [48]	Rapid realist review with 15 studies. Context Mechanism Outcomes configurations were extracted and associated with clinical effectiveness, acceptability and safety	Remote reviews were safe, effective and acceptable with an advantage over face to face reviews of accessibility; however, use should be guided by patient preference and professionals may require guidance on optimising their use

### Develop detailed intervention design

A comprehensive implementation strategy was then defined. A full description of the prototype strategy including mapping to both COM-B and iPARIHS constructs is available in supplementary file 2 and a summary description is provided in Table 2.

**Table 2:** Overview of the IMP^2^ART Implementation Strategy

IMP^2^ART Implementation Strategy	
Patient resources	• Invitation Letters • Waiting Room Posters • Patient website • SMS messages
Professional resources	• Online Team Education • Online Individual Study • Professional website
Organisational resources	• Review Template • Audit & Feedback – annual and monthly
Facilitation	• Dedicated Facilitator • Team workshop & Plan • 12 month access to support

The aim was for each practice randomised to the implementation group to be allocated a facilitator who would work with the practice over a period of 12 months to embed and tailor supported self-management using those implementation strategies most suitable for the practice. The lead facilitator and three facilitator colleagues were all experienced nurses with respiratory training and prior experience of facilitation. IMP^2^ART-specific training was provided for facilitators to enhance facilitation skills relevant to the context and included modelling by the lead facilitator, and joint supervision and peer support where facilitators could share and learn from experiences throughout the delivery period.

All practices had a 1-h facilitated face-to-face workshop to which all practice team members were invited. This workshop served to introduce IMP^2^ART, its objectives and the IMP^2^ART resources. All practices were introduced to a range of patient materials including accessible action plans, a website with patient (and healthcare professional resources) with signposting to established sites such as Asthma UK, patient invitation letters to reviews and posters for use in the practice. A patient-centred template to structure asthma reviews, specifically designed to address the patient agenda as well as that of the healthcare professional [49] was uploaded to all practice systems prior to the workshop. All practices also received an audit and feedback report of their previous year’s asthma management including the number of action plans provided, reviews conducted and at risk individuals [50]. Within the workshop, a whole team approach was endorsed and a team education module (team awareness module: module 1) introduced to reinforce this. Healthcare professionals in the practice who delivered care to people with asthma were encouraged to undertake an on-line 1-h individual supporting self-management education module (individual study module: module 2) [39]. The workshop was then completed with the practice team jointly setting key tailored goals for implementing the IMP^2^ART strategy in the coming months. Each practice then received regular facilitator contacts and support over the next year and monthly and annual audit and feedback reports for 2 years. At the end of 1 year, a closing workshop with the facilitator was conducted that reflected on progress, including review of an annual audit report and made plans for sustainability going forward. Sustainability was addressed using a problem-solving approach, considering individual practice barriers and facilitators with the implementation of supported self-management, as well as setting a final practice team plan of activities to take forward to support ongoing use of supported self-management.

### Pre-pilot

The pre-pilot was conducted in four practices over a period of 3 months. Two facilitators delivered all workshops face-to-face (spring/summer 2019) which were attended in total by 26 general practice staff including GPs, nurses, administrators, practice managers, healthcare assistants. A member of the IMP^2^ART team (KM) also attended two of the workshops as an observer and made field notes. There was considerable variability in attendance, the amount of time available for workshops (10–60 min) as well as technical challenges. It was clear that it was not possible to run the team education module in full and set a team action plan as envisaged, within the time available. Facilitators (in agreement with the observing team members) proposed that the workshop be used as a free-flowing session, picking up points from the team education module but focusing more on development of the team plan. This was rated a ‘must have’ according to MoSCoW criteria [40] and was therefore a critical change. A sequence of emails to highlight importance of whole team attendance at the workshop were also developed and considered a ‘should have’.

The pre-pilot also tested upload of the template which was well received with only minor changes. Audit and feedback were also reported as clear and easy to use.

### Finalising the implementation strategy and programme theory

Review of the implementation components at the onset of the COVID-19 pandemic suggested that no changes needed to be made to the practice templates. The annual audit and feedback reports had a section added which covered COVID-19 (e.g. vaccination status, risk) and patient letters and invitations referred to remote appointments. Changes were however felt relevant for both the patient and healthcare professional website, the individual education module and the facilitation approach. These were primarily addition of new materials and are shown in supplementary file 2 in italics to reflect ‘COVID-19’ modifications. The most significant change consequent to the changing context was moving from in-person facilitation to a remote facilitation, with the workshop delivered by Microsoft Teams. Subsequent planned contacts between facilitators and practices were almost wholly remote.

In addition, a range of resources to support healthcare professionals delivering remote reviews was provided and drew on a realist review that was completed by the team which found that remote reviews for asthma self-management were safe, acceptable and clinically effective [48]. There were no significant changes made to the implementation strategy following the internal pilot.

**Fig. 3:**
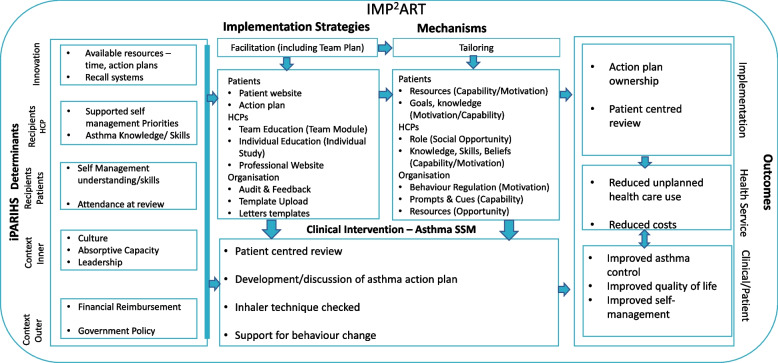
IMP^2^ART Implementation Logic Model. The implementation strategy guided by iPARIHS determinants and acting through COM-B mechanisms is hypothesised to increase implementation of supported self-management with consequent impact on health outcomes

Figure 3 shows the final logic model used to guide the implementation strategy designed in line with recommendations [51]. This served as a basis for the process evaluation.

## Discussion

Implementation of interventions in primary care provides a particular challenge given the multiple systems with and within which they work [52]. However, given their accessibility and that they are often the first point of contact with the health service for patients, ensuring implementation of evidence-based practice in this context is a priority for healthcare systems worldwide [15]. The implementation programme presented here (IMP^2^ART) describes a model for increasing supported self-management in asthma. Given the central role of supported self-management to many chronic conditions, there is considerable potential for generalisability from the learning in this project.

As described in this paper, the IMP^2^ART model is based on extensive use of theory (including from multiple disciplines), the evidence base and lived experience. We have followed guidance for development of interventions and described this in line with recommendations for both intervention development [53] and implementation research [54]. Considerable work was conducted to identify key guiding principles, appropriate theory, and feasibility work in the format of a pre-pilot where aspects of the strategy were integrated and tested together. The latter has been recommended previously as key to understanding not only the independent delivery of specific components but also the interaction of elements and whether there is conflict or synergy between elements of the overall strategy [55]. An example of the benefits of this approach was identification of a tension between available resources (e.g. time, technological access) and amount of content to be covered in the facilitator workshop. Having identified this tension, changes were made to the implementation strategy. Omitting the pre-pilot phase would have meant changes would have had to be made after the internal pilot phase of the trial, and the 12 pilot practices could not have been included in the main trial, with implications for trial recruitment and power.

In developing the programme of work, we have described how different theories were integrated to form a platform from which analysis and greater depth of understanding can occur. The use of theory has been described by many as a critical element in implementation science and its specification will enable us to consider multiple levels of change that are likely to be needed for successful implementation [22, 34]. By incorporating both the COM-B and iPARIHS frameworks, we bring together the disciplines of health psychology and implementation science and work towards recommendations [56] to recognise the synergy of these disciplines. Also, by using this integrated model as a basis for evaluation with iterative feedback, we are participating in the act of not only describing, but theorising, as recommended by Kislov et al. [22]. Further, we respond to the call for further conceptualisation of the recipient domain in iPARIHS [57] and will use our process evaluation to empirically test our conceptualisation.

### Recommendations for evaluation

An important objective in our comprehensive design and description of the implementation strategy is that not only should it increase likelihood of effectiveness, but it should enable evaluative efforts to understand the mechanisms of action. The hypothesis around the central role of facilitation as the active ingredient of implementation, with recipients actioning change when they have developed sufficient capability, opportunity and motivation, and context playing a significant moderating factor across all elements will be examined in the process evaluation. The process evaluation is carefully mapped to the underpinning theory and will ask questions such as ‘Do those practices that engage with facilitation differently demonstrate greater change? Does variation in capability, opportunity and motivation at the individual and team level act as a barrier (or facilitator) to change, and are changes in leadership or culture significant in that change? Informed by such data, a revised programme theory with recommendations for sustainable implementation in primary care practice will be made.

An additional critical element to understanding the utility of the implementation strategy is assessment of the fidelity with which the strategy is delivered. Fidelity relates to the extent an intervention is delivered as intended; and can be described in terms of five domains (i) study design, (ii) provider delivery, (iii) treatment delivery, (iv) treatment receipt and (v) treatment enactment [58, 59]. Implementation interventions need to balance the need for fidelity with that for adaptation and it has been proposed that these concepts are intrinsically linked [60]. StaRI further specifies that reporting of implementation studies should include fidelity to implementation strategy as planned, and adaptation to suit context and preference [54] and there have been recent frameworks published for the reporting of this [61, 62]. It is a prerequisite therefore that there is a clear description of the implementation strategy, core elements and conceptualisation of how adaptation is envisaged. The detailed description of IMP^2^ART provided here allows for this level of analysis to take place.

The occurrence of the COVID-19 pandemic during the development phase of our implementation strategy acted as an ‘in vivo’ test for how our strategy could be influenced by external context and adapted appropriately. The strategy was able to be adapted without compromising the core components and it is a strength that IMP^2^ART is now compatible with multiple modes of consultation given the post-COVID norm of offering remote healthcare. The extent that remote delivery, particularly of facilitation, is successful is however yet to be ascertained but will be explored as part of the process evaluation.

### Strengths and limitations

It is a strength of our implementation strategy that its development has followed best practice guidance and each step has been explicitly described and made publicly available [39, 49, 50]. By pre-piloting the strategy, we got a strong sense of how the components worked together and we were able to make refinements before entering the internal pilot phase of the study.

One limitation of our approach was that we did not choose a theory based on a tool such as the implementation Theory and Selection Tool (T-CAST) [63] or strictly in accordance with guides such as Lynch et al. [64]. Using such tools or guidance may have led to selection of a different theory and/or a more systematic approach to theory selection. Although not following a documented approach, we did take a systematic and informed approach where decisions were made within a multidisciplinary team with considerable expertise in a range of areas including theory, practice and methodology. Decisions on theory were taken as a team using a consensus approach and there with an openness to change if this was deemed appropriate.   

## Conclusion

The IMP^2^ART study is a large UK cluster randomised implementation trial aiming to embed supported asthma self-management in primary care practice. We have described a robust process for design of the implementation strategy including development of a programme theory. We propose an advancement in the field by way of integration of implementation science theory (iPARIHS) and behaviour change theory (COM-B) which will be evaluated in a comprehensive process evaluation.

### Supplementary Information

**Additional file 1:** **Supplementary File 1.** Preliminary theoretical Mapping of the iPARIHS constructs and the Theoretical Domains Framework (TDF) and Capability Opportunity Motivation-Behaviour (COM-B) framework.

**Additional file 2:** **Supplementary File 2.** Full description of the IMP^2^ART Implementation Strategy with mapping to IPARIHS and COM-B

## Data Availability

All data generated or analysed during this study are included in the published articles [23, 27, 28,30,39] and their supplementary information files.

## Abbreviations

COM-B: Capability Opportunity Motivation Behaviour
HCP: Health Care Professional
IMP2ART: IMPlementing IMProved Asthma self-management as RouTine
iPARIHS: Integrated Promoting Action Research in Healthcare Services
MRC: Medical Research Council
NIHR: National Institute of Health and care Research
UK: United Kingdom

## References

[1] Gibson PG, Powell H, Wilson A, Abramson MJ, Haywood P, Bauman A, et al*.* Self-management education and regular practitioner review for adults with asthma. Cochr Database Syst Rev. 2003;(1):CD0011172. 10.1002/14651858.CD001117. Accessed 30 Oct 2023. 10.1002/14651858.CD001117 12535399

[2] D'Souza W, Crane J, Burgess C, Te Karu H, Fox C, Harper M (1994). Community-based asthma care: trial of a "credit card" asthma self-management plan. Eur Resp Jn.

[3] Pinnock H, Parke HL, Panagioti M, Daines L, Pearce G, Epiphaniou E, Bower P, Sheikh A, Griffiths CJ, Taylor SJC, for the PRISMS group (2017). Systematic meta-review of supported self-management for asthma: a healthcare service perspective. BMC Med.

[4] Hodkinson A, Bower P, Grigoroglou C (2020). Self-management interventions to reduce healthcare utilisation and improve quality of life among patients with asthma: a systematic review and network meta-analysis. BMJ.

[5] British Thoracic Society, Research Unit of the Royal College of Physicians of London, King's Fund Centre. National Asthma Campaign (1990). Guidelines for management of asthma in adults: I-chronic persistent asthma. BMJ..

[6] Global Initiative for Asthma. Global Strategy for Asthma Management and Prevention, 1995. https://ginasthma.org/wp-content/uploads/2019/01/1995-GINA.pdf (Accessed Dec 2022).

[7] Renwick L, Cummella A, Wilson-Edwards H. Fighting back. Asthma + Lung UK 2022 https://www.asthmaandlung.org.uk/sites/default/files/Fighting%20back_V3.pdf (Accessed Feb 2023)

[8] Wiener-Ogilvie S, Pinnock H, Huby G, Sheikh A, Partridge MR, Gillies J (2007). Do practices comply with key recommendations of the British asthma guideline? if not, why not?. Prim Care Respir J.

[9] Stallberg B, Lisspers K, Hasselgren M, Janson C, Johansson G, Svardsudd K (2009). Asthma control in primary care in Sweden: a comparison between 2001 and 2005. Prim Care Respir J.

[10] Centers for Disease Control and Prevention (2013). Asthma Facts—CDC’s National Asthma Control Program Grantees.

[11] Sulaiman N, Aroni R, Thien F, Schattner R, Simpson P, Del Colle E, Wolfe R, Abramson M (2011). Written Asthma Action Plans (WAAPs) in Melbourne general practices: a sequential mixed methods study. Prim Care Respir J.

[12] Newby C, Wright N, Eldridge S, Morrow S, Vince-Lawer E, Appiagyei F, Hjelmbjerg T, Skinner D, Price D, Taylor S, Pinnock H (2017). Estimating exacerbation rates from routine UK primary care data: an exploratory validation from the IMP2ART programme. Eur Resp J..

[13] British Thoracic Society/Scottish Intercollegiate Guideline Network. British Guideline on the Management of Asthma. 2019update. Available from http://www.sign.ac.uk/sign-153-british-guideline-on-the-management-of-asthma.html (Accessed Dec 2022).

[14] Murray CJ (2020). Global burden of 369 diseases and injuries in 204 countries and territories, 1990–2019: a systematic analysis for the global burden of disease study 2019. Lancet.

[15] World Health Organisation. Asthma: Key Facts. 2022 https://www.who.int/news-room/fact-sheets/detail/asthma (Accessed 9 Sept 2022)

[16] National Institute for Clinical Excellence. Asthma: diagnosis, monitoring and chronic asthma management. NICE guideline NG80. 2017. Last updated 2021. 34101394

[17] Global Initiative for Asthma. Global Strategy for Asthma Management and Prevention, 2023. https://ginasthma.org/2023-gina-main-report/ (Accessed June 2023).

[18] Taylor SJC, Pinnock H, Epiphaniou E, Pearce G, Parke H (2014). A rapid synthesis of the evidence on interventions supporting self-management for people with long-term conditions. (PRISMS Practical Systematic Review of Self-Management Support for long-term conditions). Health Serv Deliv Res.

[19] Pinnock H, Epiphaniou E, Pearce G (2015). Implementing supported self-management for asthma: a systematic review of implementation studies. BMC Med.

[20] NHS England. National bundle of care for children and young people with asthma 2021. https://www.england.nhs.uk/publication/national-bundle-of-care-for-children-and-young-people-with-asthma (Accessed Feb 2023)

[21] Birken SA, Powell BJ, Presseau J (2017). Combined use of the Consolidated Framework for Implementation Research (CFIR) and the Theoretical Domains Framework (TDF): a systematic review. Implementation Sci.

[22] Kislov R, Pope C, Martin GP (2019). Harnessing the power of theorising in implementation science. Implementation Sci.

[23] McClatchey K, Hammersley V, Steed L et al. IMPlementing IMProved Asthma self-management as RouTine (IMP^2^ART) in primary care: study protocol for a cluster randomised controlled implementation trial protocol. Trials. 2023;24:252. 10.1186/s13063-023-07253-9. 10.1186/s13063-023-07253-9 PMC10068707 37013577

[24] Skivington K, Matthews L, Simpson SA, Craig P, Baird J, Blazeby JM (2021). A new framework for developing and evaluating complex interventions: update of Medical Research Council guidance. BMJ.

[25] Yardley L, Arden-Close E, Muller I (2015). The person-based approach to enhancing the acceptability and feasibility of interventions. Pilot Feasib Stud.

[26] Pearce G, Parke H, Pinnock H, Epiphaniou E, Bourne CLA, Sheikh A, Taylor SJC (2016). The PRISMS taxonomy of self-management support: derivation of a novel taxonomy and initial testing of utility. J Health Serv Res Policy.

[27] McCleary N, Andrews A, Captieux M (2018). IMP^2^ART systematic review of education for healthcare professionals implementing supported self-management for asthma. Npj Prim Care Respir Med.

[28] Cane J, O’Connor D, Michie S (2012). Validation of the theoretical domains framework for use in behaviour change and implementation research. Implement Sci.

[29] Ivers N, Jamtvedt G, Flottorp S, Young JM, Odgaard-Jensen J, French SD, O'Brien MA, Johansen M, Grimshaw J, Oxman AD. Audit and feedback: effects on professional practice and healthcare outcomes. Cochr Database Syst Rev. 2012;(6):CD000259. 10.1002/14651858.CD000259.pub3. Accessed 30 Oct 2023. 10.1002/14651858.CD000259.pub3 PMC11338587 22696318

[30] Morrow S, Daines L, Wiener-Ogilvie S (2017). Exploring the perspectives of clinical professionals and support staff on implementing supported self-management for asthma in UK general practice: an IMP^2^ART qualitative study. npj Prim Care Respir Med.

[31] Morrissey M, Shepherd E, Kinley E, McClatchey K, Pinnock H. Effectiveness and perceptions of using templates in long-term condition reviews: a systematic synthesis of quantitative and qualitative studies. Brit J Gen Pract 2021; BJGP.2020.0963. 10.3399/BJGP.2020.0963 10.3399/BJGP.2020.0963 PMC8321439 33690148

[32] Ramanathan A, Sheringham J. A rapid review of the influence of contextual factors on innovation in self-management strategies for primary-care based asthma management. PCRS 2019

[33] O'Cathain A, Croot L, Duncan E (2019). Guidance on how to develop complex interventions to improve health and healthcare. BMJ Open.

[34] Nilsen P (2015). Making sense of implementation theories, models and frameworks. Implementation Sci.

[35] Michie S, Atkins L, West R. The behaviour change wheel : a guide to designing interventions 2014.

[36] Public Health England. Behaviour change: guides for national and local government and partners, 2020.

[37] Greenhalgh T (2008). Roles of routines in collaborative work in healthcare organisations. BMJ.

[38] Daines L, Morrow S, Wiener-Ogilvie S (2020). Understanding how patients establish strategies for living with asthma: IMP2ART qualitative study. Br J Gen Pract.

[39] McClatchey K, Marsh V, Steed L (2022). Developing a theoretically informed education programme within the context of a complex implementation strategy in UK primary care: an exemplar from the IMP*2*ART trial. Trials.

[40] Clegg D, Barker R. Case Method Fast-Track: A RAD Approach. Addison-Wesley. ISBN 978–0–201–62432–8;1994

[41] Green MA, McKee M, Katikireddi SV (2022). Remote general practitioner consultations during COVID-19. Lancet Digit Health.

[42] Health Foundation, Nuffield Trust. The remote care revolution during Covid-19. https://www.nuffieldtrust.org.uk/files/2020-12/QWAS/digital-and-remote-care-in-covid-19.html#4 Date last accessed: 29 Mar 2023

[43] NHS Confederation, British Medical Association (2003). New GMS Contract 2003: Investing in General Practice.

[44] Greenhalgh T, Robert G, Macfarlane F, Bate P, Kyriakidou O (2004). Diffusion of innovations in service organizations: systematic review and recommendations. Milbank Q.

[45] Mannion R, Davies H. Understanding organisational culture for healthcare quality improvement. BMJ 2018; 363. 10.1136/bmj.k4907 10.1136/bmj.k4907 PMC6260242 30487286

[46] Harvey G, Kitson A (2015). PARIHS revisited: from heuristic to integrated framework for the successful implementation of knowledge into practice. Implementation Sci.

[47] McClatchey K, Jackson T, Delaney B, Barat A, Morgan N, Pinnock H, Chan AHY (2021). COVID-19 information for people living with asthma: A rapid review of publicly available information guidance. J Allergy Clin Immunol.

[48] Kinley, E, Skene, I, Steed, L, et al. Delivery of supported self-management in remote asthma reviews: A systematic rapid realist review. Health Expect. 2022; 1-15. 10.1111/hex.13441 10.1111/hex.13441 PMC9327809 35411670

[49] McClatchey K, Sheldon A, Steed EA, Sheringham J, Appiagyei F, Price D, Hammersley V, Taylor SJC, Pinnock H, for the IMP^2^ART Programme Group. Development of a patient-centred electronic review template to support self-management in primary care. BJGP Open 3 March 2023; BJGPO.2022.0165. 10.3399/BJGPO.2022.0165 10.3399/BJGPO.2022.0165 PMC10354399 36868789

[50] McClatchey K., Sheldon A., Steed L., Sherringham J. Appiagyei F et al. Development of theoretically informed audit and feedback: an exemplar from a complex implementation strategy to improve asthma self-management in UK primary care. Jn Eval Clin Prac. 10.1111/jep.13895 10.1111/jep.13895 37438918

[51] Smith JD, Li DH, Rafferty MR (2020). The implementation research logic model: a method for planning, executing, reporting, and synthesizing implementation projects. Implementation Sci.

[52] Prathivadi P, Buckingham P, Chakraborty S (2022). Implementation science: an introduction for primary care. Fam Pract.

[53] Duncan E, Rousseau N, Croot L (2020). Guidance for reporting intervention development studies in health research (GUIDED): an evidence-based consensus study. BMJ open..

[54] Pinnock H, Barwick M, Carpenter C (2017). Standards for Reporting Implementation Studies (StaRI) statement. BMJ.

[55] Steed L, Heslop-Marshall K, Sohanpal R (2021). Developing a complex intervention whilst considering implementation: the TANDEM (Tailored intervention for ANxiety and DEpression Management) intervention for patients with chronic obstructive pulmonary disease (COPD). Trials.

[56] Presseau J, Byrne-Davis LMT, Hotham S (2022). Enhancing the translation of health behaviour change research into practice: a selective conceptual review of the synergy between implementation science and health psychology. Health Psychol Rev.

[57] Duan Y, Iaconi A, Wang J (2022). Conceptual and relational advances of the PARIHS and i-PARIHS frameworks over the last decade: a critical interpretive synthesis. Implementation Sci.

[58] Bellg AJ, Borelli B, Resnick B (2004). Enhancing treatment fidelity in health behaviour change studies: best practices and recommendations from the NIH Behavior Change Consortium. Health Psychol..

[59] Borrelli B (2011). The assessment, monitoring, and enhancement of treatment fidelity in public health clinical trials. J Public Health Dent..

[60] Pérez D, Van der Stuyft P, Zabala MC, Castro M, Lefèvre P (2016). A modified theoretical framework to assess implementation fidelity of adaptive public health interventions. Implement Sci.

[61] Miller C, Barnett ML, Baumann AA (2021). The FRAME-IS: a framework for documenting modifications to implementation strategies in healthcare. Implementation Sci.

[62] Kirk MA, Moore JE, Wiltsey Stirman S (2020). Towards a comprehensive model for understanding adaptations’ impact: the model for adaptation design and impact (MADI). Implementation Sci.

[63] Birken SA, Rohweder CL, Powell BJ (2018). T-CaST: an implementation theory comparison and selection tool. Implementation Sci.

[64] Lynch EA, Mudge A, Knowles S (2018). “There is nothing so practical as a good theory”: a pragmatic guide for selecting theoretical approaches for implementation projects. BMC Health Serv Res.

