# Supplementary material for: IMP2ART: development of a multi-level programme theory integrating the COM-B model and the iPARIHS framework, to enhance implementation of supported self-management of asthma in primary care
Source: Implement Sci Commun. 2023 Nov 13;4:136. doi: 10.1186/s43058-023-00515-2 (PMC10644643; doi:10.1186/s43058-023-00515-2)
Supplement: Supplementary file 1 — Additional file 1: Supplementary File 1. Preliminary theoretical Mapping of the iPARIHS constructs and the Theoretical Domains Framework (TDF) and Capability Opportunity Motivation-Behaviour (COM-B) framework. [file 43058_2023_515_MOESM1_ESM.docx]

Supplementary File One: Preliminary theoretical Mapping of the iPARIHS constructs and the Theoretical Domains Framework (TDF) and Capability Opportunity Motivation-Behaviour (COM-B) framework.

**Innovation – needs to be a process of aligning external explicit evidence with local priorities to enhance compatibility of proposed change**

Underlying knowledge sources (evidence that supported self-management (SSM) improves outcomes)

Clarity (clear what SSM is – need to understand what SSM means for each practice?)

Degree of fit with existing practice and values - compatibility or contestability (what is fit, where will changes need to be made – start with current practices and evolve)

Usability (can it be completed the same time as normal practice)

Relative advantage (how will this benefit the practice/practice staff/patient)

Trialability (what can be learned from trying out the strategies)

Observable results (patients come back with better control)

**Recipient – the people who are affected by and influence implementation at both individual and collective team level**

Motivation (Reflective Motivation)

Values and Belief (Reflective motivation TDF – Belief about consequences)

Goals (Reflective motivation TDF - Goals)

Skills and Knowledge (Psychological and Physical capability – TDF knowledge, physical skills, interpersonal skills)

Time, resources, support (Practical opportunity – TDF Environmental context and resources, Social influences)

Local opinion leaders (Social opportunity – TDF Social influences)

Collaboration and Team work (Social opportunity TDF Social influences, Social/professional role and identity)

Existing networks (Social opportunity)

Power and authority (TDF Social/professional role and identity)

Presence of boundaries (TDF Social/professional role and identity)

It is assumed that actions from facilitator and implementation strategies will influence health care professionals/team which impact patients. Key theoretical mechanisms and behaviour change techniques through which change is anticipated to occur are highlighted.

| COM-B Element | TDF | Recipient  Patient Behaviour | Recipient HCP (Practice Nurse – PN /team)  *Behaviour Change Technique* | Facilitation and implementation strategies  *Behaviour Change Techniques* |
| --- | --- | --- | --- | --- |
| Physical Capability | Physical Skills | To use an inhaler | 4.1 instruction on behaviour  6.1 demonstration of behaviour |  |
| Psychological Capability | Knowledge  Cognitive & interpersonal skills  Memory, attention and decision making  Behavioural regulation | What is an Action Plan  Social skills to discuss asthma including with HCPs.  Decision making on when to change treatment  Patients encouraged to monitor so can see impact of behavior on symptoms | How to complete and individualize Action Plans (PN) *1.4 Action Planning,*  *1.5 Review Behavioural Goals,*  *4.1 Instruction on behaviour*  Communication skills to support behavior change (PN)  *1.2 Problem Solving,*  *3.1 Social support*  Autonomy & team support for PN to support advice on medications  6.3  Feedback on SSM improves outcomes  *2.7 Feedback on Outcomes of Behaviour* | Education Module 2- completing Action Plan  *4.1 instruction on behaviour*  *6.1 demonstration of behaviour*  *8.1 behavioural practice*  Education Module 2 – a Motivational Interviewing style to communication  *5.1 information on health consequences*  *6.1 demonstration of behaviour*  *8.1 behavioural practice*  Education Module1 -Agreed role and support from team  *6.3 Information about others approval*  Audit & Feedback Reports  *1.6 – discrepancy between current behaviour and goal*  *6.2 social comparison* |
| Practical Opportunity | Environmental context and resources | Have sufficient appointments for review  Access to resources e.g. Action Plan | Regular Reviews set up and  Time for consultation  Have resources i.e. Action Plans to hand | Patients sent review letters, access to website  Facilitator to provide team with the range of resources |
| Social Opportunity | Social influences | HCP and health team reinforce self-management as important | Practice supports SM in time and attitude | Education Module 1 facilitate whole team approach  *9.1 credible source* |
| Reflective Motivation | Prof/social role and identity  Beliefs about capability  Beliefs about consequences  Goals | Confidence they should and can play role in self management  Expectation of what level of control can be achieved.  Goals set | PN and whole team see role within SSM  PN confidence to let patient self manage  PN believes patients can self manage  PN believes SSM will lead to improved outcomes  *5.1 information on health consequences*  *5.3 social support*  *2.7 Feedback on outcome of behaviour* | Education Module 1 facilitate whole team approach  Workshop and Education Module 2 to support PN in capability and self efficacy  Case studies to influence belief about consequences  *5.1 information on health consequences*  *5.2 salience of consequences*  *5.3 social support*  Facilitation Workshop and Team Plan  *1.1 goal setting behaviour* |
| Automatic Motivation | Reinforcement  Emotion | Support to perform positive habits (e.g. take inhaler at regular time), break unhelpful habits | Habitually provide action plan and have person-centred consultations  *1.2 problem solving*  *8.1 behavioural practice,*  *8.3 habit formation* | Template (prompts repeated behavior until a habit)  *7.1 prompts* |
