# Supplementary material for: IMP2ART: development of a multi-level programme theory integrating the COM-B model and the iPARIHS framework, to enhance implementation of supported self-management of asthma in primary care
Source: Implement Sci Commun. 2023 Nov 13;4:136. doi: 10.1186/s43058-023-00515-2 (PMC10644643; doi:10.1186/s43058-023-00515-2)
Supplement: Supplementary file 2 — Additional file 2: Supplementary File 2. Full description of the IMP2ART Implementation Strategy with mapping to IPARIHS and COM-B [file 43058_2023_515_MOESM2_ESM.docx]

Supplementary File 2: Full description of the IMP^2^ART Implementation Strategy with mapping to IPARIHS and COM-B

| Target Group | Strategy **(bold is core content)** | Description | iPARIHS Elements | COM-B (TDF) Element* |
| --- | --- | --- | --- | --- |
| Patient |  |  |  |  |
|  | Asthma review invitation letters/SMS messages | Template asthma review invitation letters (including letters for annual reviews, missed review appointments, reviews following unscheduled care), that highlight the importance of reviews and asthma action plan ownership.  *Invitation letters adapted to include remote options e.g. telephone/video-call.* | Innovation  Individual Recipient (patient) | Reflexive Motivation (Belief about Consequences) |
|  | **Patient content of Living with Asthma Website** | A range of online resources for people living with asthma (e.g. action plans, information about triggers etc.) have been collated on patient-facing pages of the Living with Asthma website.  *Informed by a review of existing information about COVID-19, added tailored information for those living with asthma, and remote asthma review information.* | Innovation  Individual Recipient (patient) | Psychological Capability  (Knowledge)  Physical Opportunity  (Environmental Context/Resources)  Reflexive Motivation  (Belief about consequences) |
|  | Waiting room posters (electronic and hard copy) | Waiting room posters for practices highlighting the importance of self-management and asthma action plans, and encouraging patients to speak to staff about these.  *No Change* | Innovation  Recipient (individual patient)  Team Recipient  (professional) | Reflexive Motivation (Belief about consequences) |
| Professional |  |  |  |  |
|  | **Module 1: Team module** | A short introductory online module for the whole-practice team (e.g. administrative staff, nurses, GPs) to raise awareness of supported asthma self-management and the importance of team working. This interactive module will be introduced in the one-hour IMP^2^ART workshop, to facilitate discussion about IMP^2^ART strategies that the practice could adopt to support/embed self-management. Goals are set and summarised as the practice ‘team plan’.  *No change to the module, though the IMP^2^ART workshop was shifted to on-line.* | Innovation  Team Recipient  (professional) | Social Opportunity  (Social influences)  Reflexive Motivation (Role, Goals) |
|  | **Module 2: Individual study** | An in-depth online module for the individual(s) in the practice most involved with delivering asthma care. The module is designed for 60 minutes of independent study, and aims to enable healthcare professionals to support effective self-management with confidence, and be motivated to adopt the IMP^2^ART resources identified in their practice ’team plan’.  *Content was added to the module that covered effective remote consultation skills [39].* | Innovation  Recipient (Individual professional) | Psychological Capability  (Knowledge, skills)  Reflexive Motivation  (Belief about capabilities) |
|  | Professional content of Living with Asthma website | A range of online resources for healthcare professionals (e.g. action plans, patient information for use in reviews etc.) have been collated on the professional pages of the Living with Asthma website  *Added information and resources related to providing remote asthma reviews* | Innovation  Individual Recipient (professional) | Physical Capability  Psychological Capability  (Knowledge) |
| Organisational |  |  |  |  |
|  | **Asthma Review Template** [31,49] | An asthma review template that will be embedded in practice systems (EMIS, SystmOne, Vision, Microtest) for use in asthma reviews. Templates will be ‘QOF-compliant’ [43], patient-centred, highlight action plan provision, and link to the IMP^2^ART patient website.  *No change.* | Innovation  Recipient (Individual professional/patient)  Context | Psychological Capability (Memory)  Physical Opportunity  (Environmental context and resources) |
|  | **Audit & Feedback [50]** | Annual audit reports focusing on supported self-management will be provided at baseline, 12, and 24 months. Brief monthly reports will be delivered to practices for the duration of their participation in the trial, focusing on a summary of unscheduled care, patients reviewed, and action plan provision compared to an OPC database average. The email containing the monthly report will include a ‘top tip’. Reports allow data to be de-anonymised by the practices, so that ‘at-risk’ patients can be identified and invited for review.  *Annual audit and feedback report updated to include a section on COVID-19: number/% of patients considered at high risk of complications due to COVID-19; number/% of patients with confirmed or suspected COVID-19; patient COVID-19 vaccination status (number/%).* | Innovation  Recipient  (Team - professional)  Context | Psychological capability  (Knowledge, Behavioural Regulation) |
|  | **Facilitation** | One hour introductory workshop plus up to 10 hours of contacts over following 12 months and exit workshop. Delivered by trained expert respiratory nurse.  *Transition to remote delivery of facilitation mainly through MS Teams meetings* | Recipient (Team-professional)  Context | Social Opportunity  (Social Influences)  Reflexive Motivation  (Goals) |

Details in italics reflect elements added after COVID-19, iPARIHS – integrated Promoting Action on Research Implementation in Health Services, COM-B – Capability, Opportunity, Motivation – Behaviour Framework. * Coding is not exhaustive but rather reflects the most prominent elements through which this strategy is anticipated to work.
